# Eliciting dose is associated with tolerance development in peanut and cow’s milk allergic children

**DOI:** 10.1186/s13601-019-0298-z

**Published:** 2019-11-06

**Authors:** C. Nitsche, C. D. Westerlaken-van Ginkel, B. J. Kollen, A. B. Sprikkelman, G. H. Koppelman, A. E. J. Dubois

**Affiliations:** 1 University of Oldenburg, Oldenburg, Germany; 2 Department of Pediatric Pulmonology and Pediatric Allergology, University Medical Center Groningen, University of Groningen, CA43, PO BOX 30.001, 9700 RB Groningen, The Netherlands; 3 GRIAC Research Institute, University Medical Center Groningen, University of Groningen, Groningen, The Netherlands; 4 Department of General Practice and Elderly Care Medicine, University Medical Center Groningen, University of Groningen, Groningen, The Netherlands

**Keywords:** Food allergy, Pediatrics, Eliciting dose, Prognosis, Atopic dermatitis

## Abstract

**Background:**

Tolerance development rates differ between food allergies. Almost all previous studies have not used the gold standard method, the double-blind, placebo-controlled food challenge (DBPCFC), which may affect the reported prevalence rates. Little is known about the association of the eliciting dose (ED) obtained during the initial DBPCFC with later tolerance development.

**Methods:**

This was a retrospective, tertiary care study of children who had a positive DBPCFC to either peanut, milk or egg, and at least one follow-up food challenge (open or DBPCFC) with the same food. The association between ED and negative (tolerant) follow-up food challenge outcome was analyzed by logistic regression, with adjustment for confounders. Suspected confounders were initial DBPCFC test characteristics, atopic comorbidities and serum specific IgE (sIgE) levels.

**Results:**

In 47 peanut allergic children, tolerance developed in 27.7% (median follow-up duration of 43 months). In 80 milk (follow-up 23 months) and 55 egg (follow-up 37 months) allergic children, tolerance developed in 55.0% and 65.5%. The ED obtained during the initial DBPCFC was significantly associated with tolerance development in peanut and milk allergy, but not in egg allergy.

**Conclusion:**

Approximately 1 out of 4 children with DBPCFC confirmed peanut allergy developed tolerance, compared to more than half of the children with milk or egg allergy, respectively. Tolerance development in peanut and milk allergy is significantly associated with ED at initial DBPCFC.

**To the Editor**,

Children allergic to cow’s milk and hen’s egg show a high rate of tolerance development over time [1, 2]. Peanut allergy had been assumed to be a lifelong condition, until Hourihane et al. [3] reported a tolerance rate of 18% in peanut allergy.

The gold standard diagnostic method, the double-blind, placebo-controlled food challenge (DBPCFC) has not been consistently used to investigate the association of parameters available at diagnosis with tolerance development over time, particularly in peanut allergy.

Our aim was to analyze the association between the eliciting dose (ED) of the initial diagnostic DBPCFC with tolerance development and to identify confounding of this association by clinical parameters obtained during the initial DBPCFC.

Data was obtained from the database of the pediatric food challenge unit of the University Medical Center Groningen. Children allergic to peanut, cow’s milk or hen’s egg seen between 2001 and 2016 were included if they had a positive initial DBPCFC and at least one follow-up challenge (DBPCFC or open FC) with an interpretable outcome as part of routine clinical care. The only exclusion criterion for an initial DBPCFC was unwillingness to undergo the test, which was the case in < 2% of cases [4]. The outcome criteria and the procedure on the placebo and verum day including the dose scheme of the allergen doses given in steps were described elsewhere [5]. Complete avoidance of all forms of the tested food was advised. Follow-up was scheduled individually and the second food challenge was offered at least 1 year after diagnosis. An open FC was chosen for follow-up, if there was no evidence of tolerance or persistent food allergy after diagnosis such as uneventful unequivocal ingestion or allergic reaction to the food. We defined *tolerance* as a negative challenge (DBPCFC or open FC) after an initial positive DBPCFC. *Persistent allergy* was defined as positivity of all challenge test results during the follow-up period.

The following factors were analyzed: gender; age; coexistent eczema, asthma, allergic rhinitis, number of comorbid food allergies and the most severe previous accidental reaction (hereafter referred to as index reaction). In addition, the following parameters pertaining to the initial DBPCFC were analyzed: ED, severity of the challenge reaction and allergen specific Immunoglobulin E (sIgE) level. To determine the severity of the reaction during the initial DBPCFC and of the index reaction, we used a previously described scoring system. Symptoms of the most severe reaction during the FC, recorded by trained allergy nurses, were scored by body part (see Table 1) and summed to compute a severity index [6]. The levels of sIgE for each allergen were measured using the Pharmacia CAP System (Thermo Fisher Scientific, Uppsala, Sweden).

**Table 1:** Scoring system of reaction severity by van der Zee et al. [6]

Body part	Severity score
Skin	1
Gastrointestinal	2
Upper airway (nose, eyes, throat)	3
Lower airway (lungs)	3
Cardiovascular or neurological	3

Logistic regression analyses were performed to estimate the association between clinical factors known at the time of the initial DBPCFC and tolerance development for each food group separately. The association between ED and tolerance development was adjusted for confounding by clinical factors for each food separately. A factor changing the beta coefficient of the relationship between the ED and tolerance by 10% or more was defined as confounder. The minimum number of children in the smallest group (persistently allergic versus tolerant group) generating sufficient events per variable (EPV) in a model with sufficient power was set at five [7] (i.e. meeting the EPV rule). In order to comply with the EPV rule we were unable to test all potential factors for confounding in one model. We chose to fit the strongest confounder in the model for adjustment based on the largest deviation from the unadjusted beta coefficient. To take the lower specificity of the open FC into account, we calculated tolerance rates in the food groups including only children followed-up by DBPCFC in a separate sensitivity analysis. The Kaplan–Meier survival curve was only used to visualize the course of survival of tolerance development of the three foods over time. No log rank test was used to directly compare the individual courses. All statistical tests were performed two sided at < 0.05. IBM^**®**^ SPSS^**®**^ Statistics v23, was used for all analyses.

Of 1332 children who initially underwent a DBPCFC, 47 children allergic to peanut, 80 children allergic to cow’s milk and 55 allergic to hen’s egg met the inclusion criteria (see Fig. 1).

**Fig. 1:**
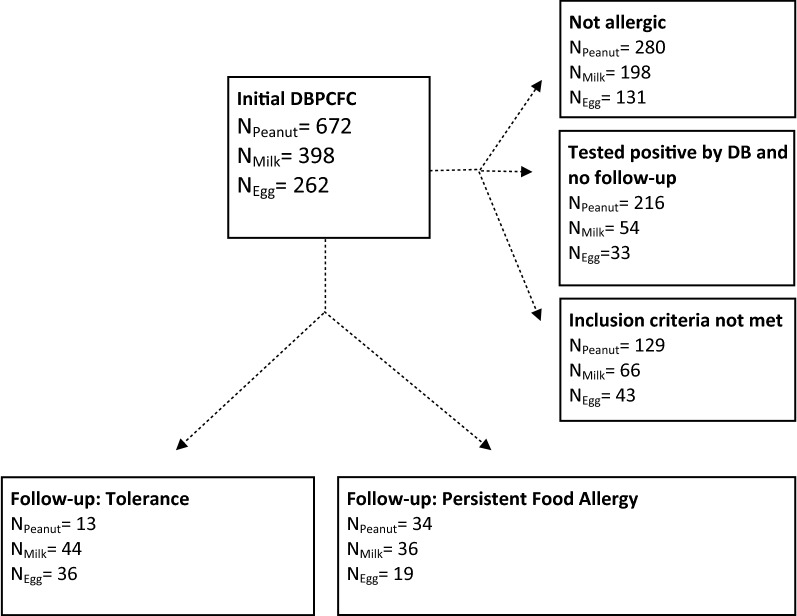
Flowchart: selection procedure of children allergic to peanut, cow’s milk or hen’s egg

Tolerance was diagnosed in 27.7% (13/47) of children allergic to peanut after a median follow-up duration of 43 months, in 55.0% (44/80) and in 65.5% (36/55) of children allergic to cow’s milk and hen’s egg after 23 and 37 months respectively (see Table 2). Eliciting dose was significantly associated with tolerance development (OR = 1.004, 95% CI 1.001–1.007, p = 0.010 for peanut; OR = 1.001, 95% CI 1.000–1.001, p = 0.001 for cow’s milk; OR = 1.001, 95% CI 1.000–1.001, p = 0.038 for hen’s egg; unadjusted). Positive history of eczema was identified as a confounder of the association between ED and tolerance development in peanut allergic children (OR = 1.005, 95% CI 1.001–1.008, p = 0.009, adjusted for eczema). No confounder was identified for the reported association for cow’s milk. In hen’s egg allergic children, the association did not reach significance after adjustment (OR = 1.000, 95% CI 1.000–1.001, p = 0.299, adjusted).

**Table 2:** Patient characteristics

Outcome	Peanut allergy				Cow’s milk allergy				Hen’s egg allergy			
	N^1^	Allergic	Tolerant	p-value	N^1^	Allergic	Tolerant	p-value	N^1^	Allergic	Tolerant	p-value
N (%)		34 (72.34)	13 (27.66)			36 (45.00)	44 (55.00)			19 (34.55)	36 (65.45)	
ED^a^ [mg/protein] (median, IQR)		228.23 [5.22;535.63)	577.97 [402.23;577.97]	*0.010*		88.38 [5.25;439.25]	1746.50 [84;2187.50]	*0.001*		89.18 [3.51;438.88]	886.54 [206.05;2184.78]	*0.038*
Follow-up in months (median, IQR)		50.50 [38;77]	43.00 [27;72]			45.00 [26;74]	23.00 [15;38]			67.00 [28;77]	37.00 [25;53]	
Age^a^ in months (median, IQR)		74.50 [51;124]	71.00 [46;98]	0.839		33.50 [14;65]	19.50 [12;38]	0.057		49.00 [22;57]	39.00 [15;58]	0.352
Sex (%), male		20 (58.82)	7 (53.84)	0.758		25 (69.44)	25 (56.82)	0.248		11 (57.89)	28 (77.78)	0.128
Asthma^a^ (%)	(33;13)	25 (75.76)	6 (46.15)	0.060		17 (47.22)	14 (31.82)	0.162	(18;35)	9 (50.00)	13 (37.14)	0.370
Eczema^a^		32 (94.12)	12 (92.31)	0.821		31 (86.11)	39 (88.64)	0.734	(19;35)	18 (94.74)	33 (94.28)	0.945
Rhinitis^a^		22 (64.71)	3 (23.08)	*0.016*	(36;42)	6 (16.67)	9 (21.43)	0.596	(18;35)	6 (33.33)	5 (15.63)	0.155
Number of diagnosed allergies to any foods (median, IQR)°		1 [1;2]	2 [1;2]	0.516		1 [1;2]	1 [1;2]	0.254		2 [1;3]	2 [1;2]	0.485
Reaction severity^a^ (median, IQR)		4 [2;5]	3 [3;4]	0.886		4 [2;6]	4 [1;6]	0.596		4 [3;7]	3 [2;4]	*0.020*
Reaction severity before diagnosis (median, IQR)		1 [0;6]	3 [0;6]	0.850		7 [4;9]	7 [4;9]	0.303		6 [2;7]	2 (0;7]	0.086
sIgE^a^ [kU/L] (median, IQR)	(29;12)	10.70 [2.58;45.90]	1.36 [.31;3.84]	0.080	(17;18)	21.65 [8.57;56.70]	2.80 [.30;6.61]	*0.001*	(17;18)	12.30 [2.51;32.20]	6.11 [1.07;10.30]	0.063

^1^N = Number of patients (allergic;tolerant); Continuous variables are presented by medians and IQRs (Inter quartile range)

^a^Measured/diagnosed at initial DBPCFC

A double-blind instead of an open follow-up challenge was performed in 24 of 47 (51.1%) peanut, in 28 of 80 (35.0%) cow’s milk and in 17 of 55 (30.9%) hen’s egg allergic children. Considering only cases followed up by DBPCFC, as many as 33.3% peanut allergic children developed tolerance. Similarly, in 75.0% of milk allergic and 76.5% of hen’s egg allergic children developed tolerance, which is higher than rates observed in previous studies [1, 2].

This is the first study in peanut allergic children using exclusively the DBPCFC for diagnosis at baseline to investigate the association between the ED at diagnosis and tolerance development. False-positive rates up to 70% reported for open FCs [8, 9] may lead to initial overestimation of food allergy and to subsequent underestimation of tolerance development.

Comparing the three food groups, a high eliciting dose was a common clinical parameter associated with tolerance in peanut and cow’s milk, but not in hen’s egg allergy when adjusted for confounding.

Despite the fact that this study was carried out using a large database, the power of analysis was still limited, leading to a limited ability to test for multiple confounders in the same association model. Although the data base includes essential patient information such as comorbid atopic diseases, it is difficult to compare these results with the general population or primary care since this research describes data of children who were referred to a tertiary care clinic with suspected food allergy. In 216 peanut, in 54 cow’s milk and in 33 hen’s egg allergic children, the outcome of only one DBPCFC was available and no follow up tests had yet been done (see Fig. 1). This was the case when the initial diagnosis was recent, if there had been clear reactions to accidental exposure during the follow up period or in rare cases, if the food has been reintroduced into the diet without medical approval. Figure 2 presents a visualization of the course of survival of tolerance development in each group over the duration of follow-up (in months). The declining curves represent the increasing number of subjects who developed tolerance over time.

**Fig. 2:**
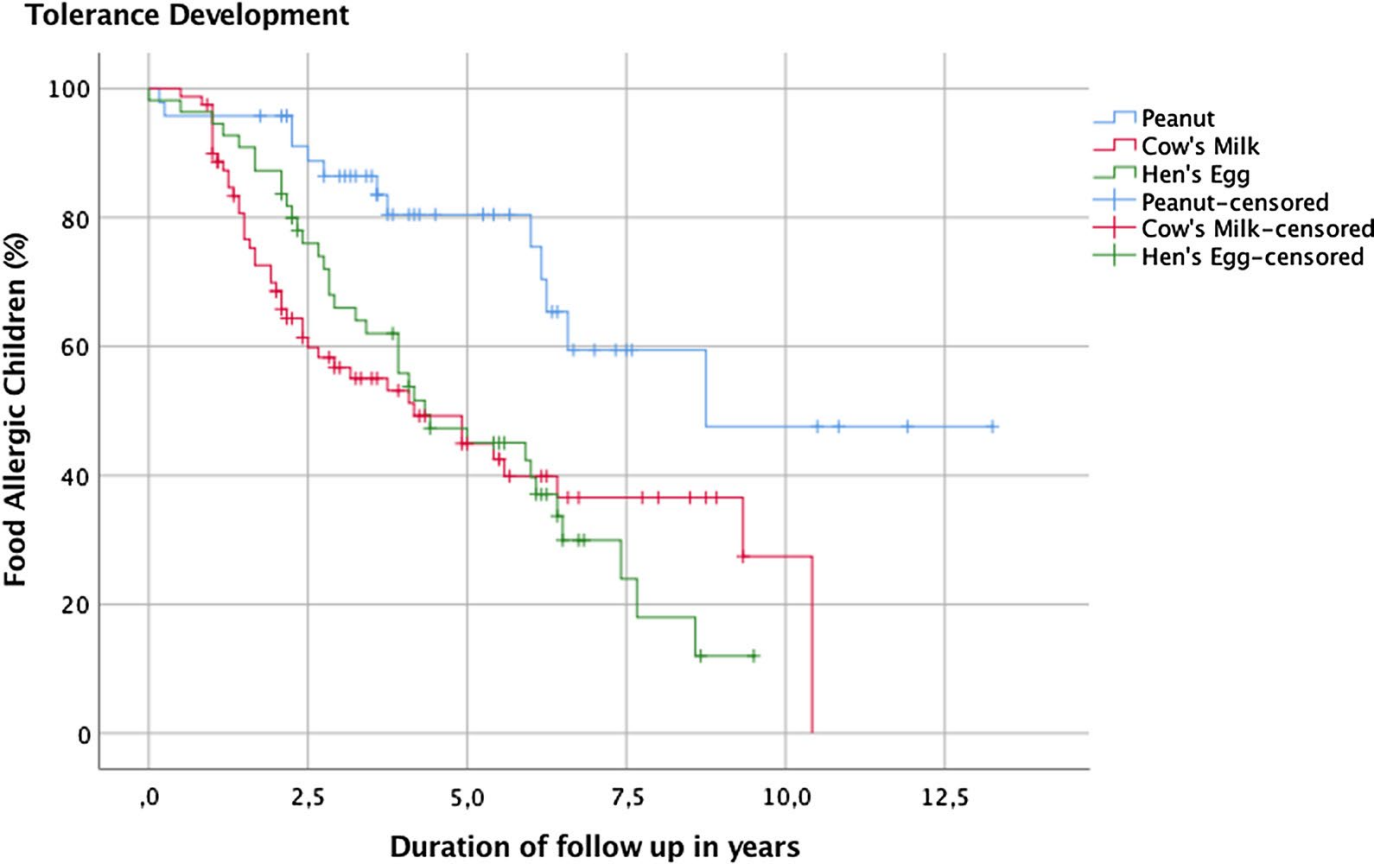
Kaplan-Meier survival curve in peanut, cow’s milk and hen’s egg allergic children

In summary, our data shows that tolerance development is common for peanut allergy as well as for cow’s milk and hen’s egg allergy, and that ED is associated with tolerance development in peanut and cow’s milk allergy. Factors associated with persistent food allergy may support clinicians to give prognosis but still cannot replace current diagnostic tools with the DBPCFC being the preferred test.

## Data Availability

The dataset analysed during the current study is not publicly available due to institutional use only but is available from the corresponding author on reasonable request.

## Abbreviations

DBPCFC: double-blind, placebo-controlled food challenge
FC: food challenge
ED: eliciting dose
sIgE: specific immunoglobulin E
